# Evaluation of a Web-Based Stress Management Program for Persons Experiencing Work-Related Stress in Sweden (My Stress Control): Randomized Controlled Trial

**DOI:** 10.2196/17314

**Published:** 2021-12-09

**Authors:** Caroline Eklund, Anne Söderlund, Magnus L Elfström

**Affiliations:** 1 Department of Physical Therapy, School of Health, Care and Social Welfare Mälardalen University Västerås Sweden; 2 Department of Psychology, School of Health, Care and Social Welfare Mälardalen University Eskilstuna Sweden

**Keywords:** behavior change, behavior medicine, internet, stress prevention

## Abstract

**Background:**

Stress is one of the most common reasons for sick leave. Web-based interventions have the potential to reach an unlimited number of users at a low cost and have been shown to be effective in addressing several health-related problems. Handling stress on an individual level is related to behavior change. To support behavioral changes in stress management, My Stress Control (MSC) was developed. The development of MSC was based on several health psychology theories and models; however, central in the development were Social Cognitive Theory, Theory of Reasoned Action, Theory of Planned Behavior, Transactional Theory of Stress and Coping, and the Transtheoretical Model and Stages of Change. MSC is a fully automated program. The program is tailored to the user’s specific needs for stress management and behavior change.

**Objective:**

In this study, we aim to conduct a randomized controlled trial to evaluate the extent to which MSC affects perceived stress in persons experiencing work-related stress.

**Methods:**

This was a randomized controlled trial with 2 arms. Study participants were recruited by visiting the worksites and workplace meetings. Participants were assigned to the intervention or wait-list group. Web-based questionnaires were used before and after the intervention to collect data. Perceived stress measured using the Perceived Stress Scale-14 was the primary outcome measurement. Analyses were conducted for both between-group and within-group changes.

**Results:**

A total of 92 participants were included in this study: 48 (52%) in the intervention group and 44 (48%) in the wait-list group. Overall, 25% (12/48) of participants in the intervention group and 43% (19/44) of participants in the wait-list group completed the postintervention assessment. There were no significant effects on perceived stress between the intervention and wait-list groups or within the groups. A small effect size (Cohen *d*=0.25) was found when comparing mean change over time on the primary outcome measure between the intervention and wait-list groups. In addition, a small effect size was found between pre- and postintervention assessments within the intervention group (Cohen *d*=0.38) as well as within the wait-list group (Cohen *d*=0.25).

**Conclusions:**

The effect of MSC on perceived stress remains uncertain. As adherence was low in the intervention group, elements or features that facilitate adherence and engagement must be further developed before firmer conclusions regarding the effect of MSC can be made.

**Trial Registration:**

ClinicalTrials.gov NCT03077568; https://clinicaltrials.gov/ct2/show/NCT03077568

## Introduction

### Background

The landscape of the work environment in Western countries is changing [1]. Traditional industries with work tasks demanding lower education levels are declining, whereas work with higher demands on education, creativity, and analytic competence is increasing. This change leads to a necessity for higher-skilled employees who are able to perform qualified work with higher demands on education and analytic competence [2]. These changes might contribute to stress being one of the main reasons for sick leave in many countries, including Sweden [3]. It has been estimated that one-fourth of the workers in Europe are at risk of developing stress-related problems [4]. In this study, stress is defined as “...a particular relationship between the person and the environment that is appraised by the person as taxing or exceeding his or her resources and endangering his or her well-being” [5].

Encouraging health-related behavior change using the internet provides the opportunity to reach out to a theoretically unlimited number of users at a lower cost than face-to-face or partly web-based solutions [6], and several web-based interventions supporting changes in different health-related behaviors have been shown to be effective [7-9]. Stress management interventions have been shown to be effective in different target groups [10-12]. Although the weaknesses of existing web-based programs for stress management have been identified, they are not often tailored or interactive and do not build on a solid theoretical framework [13].

Adherence to web applications for the management of different health-related behaviors for groups with and without various diagnoses is often low, with an average adherence of 50% [14]; this may be the result of low-grade tailoring and interactivity and program design issues. A similar pattern was observed with web-based stress management programs. A study on a web-based stress management program had a dropout rate of 40% in the intervention group [15], whereas programs with some type of coach have lower dropout rates [14,16]. One type of coach presented in an earlier study was an e-coach, providing written text within 48 hours of module completion [16]. However, this is resource demanding, and the study reported that the e-coach spent approximately 30 minutes for each person and module completion [16].

To address stress in the working population and issues related to adherence to self-management, the web application My Stress Control (MSC) [17] was developed and evaluated in a feasibility study [18]. MSC is a fully automated, interactive program tailored to the user’s individual needs for stress management based on Social Cognitive Theory (SCT), Theory of Reasoned Action, Theory of Planned Behavior, Transactional Theory of Stress and Coping, and the Transtheoretical Model and Stages of Change [17]. It was developed for persons with perceived stress who are not on sick leave and thus is used as a stress prevention intervention. The development was based on evidence within multiple fields and based on a solid theoretical framework [17] (*Methods* section).

### Objective

The hypothesis was that the newly developed web-based, self-management program built on a solid theoretical frame and incorporating evidence from multiple fields would decrease perceived stress compared with a wait-list group. Thus, the aim of this randomized controlled trial (RCT) is to evaluate the extent to which a web-based, self-management program, MSC, affected perceived stress for persons experiencing work-related stress.

## Methods

The CONSORT-EHEALTH (Consolidated Standards of Reporting Trials of Electronic and Mobile Health Applications and Online Telehealth) guidelines [19] were used for reporting this study (Multimedia Appendix 1).

### Design

This study was conducted as an RCT. After recruitment and signing informed consent forms in paper format, the participants were assigned randomly to 1 of 2 conditions: self-help stress management using the newly developed web application MSC or the wait-list control. Blinding was not applied in this study. Both participants in the intervention group and the wait-list group knew what group they were assigned to.

### Sample Size

Power for estimation of sample size was calculated using a study comparing acceptance and commitment therapy with a wait-list group and a primary outcome of perceived stress [20] measured with the Perceived Stress Scale, 14-item (PSS-14) [21]. The characteristics of the participants in both studies were expected to be similar, as both were performed in the same country. In a study by Brinkborg et al [20], the participants were divided into 2 groups: one group with lower stress and the other reporting higher stress levels [20]. The power for this study was calculated using the outcome scores from the group in the Brinkborg et al [20] study that at inclusion had stress scores <25 on the PSS-14. The estimated effect size was calculated for the expected changes in PSS-14 scores. An estimated effect size of 0.40 with power equal to 0.80 and a significance level of 0.05 gave an estimated sample size of 98 individuals in each group. With an estimated dropout rate of 20%, each group was estimated to require 118 persons for a total sample size of 236 individuals. A total of 244 persons returned the informed consent and were allocated to either the intervention or wait-list group. Thus, a number over the target sample size was recruited because it was expected that some of these persons would be excluded by MSC because of high scores on the Hospital Anxiety and Depression Scale (HADS) or low scores on PSS-14. Unfortunately, during the intervention, more persons than expected were excluded or dropped out of the study, and the sample size was not as large as expected. In the intervention group, 60% (29/48) of persons were excluded, and in the wait-list group, 86% (38/44) were excluded because of PSS or HADS scores. Overall, 52% (48/92) of persons in the intervention group and 48% (44/92) of persons in the wait-list group answered the preassessment. For the final assessment, 25% (12/48) of persons in the intervention group and 43% (19/44) of persons in the wait-list group answered.

This study was registered at ClinicalTrials.gov (NCT03077568), with a sample size of 95. This was a miscalculation where 3 persons excluded because of HADS were included in that number. These individuals did not have access to MSC and were therefore correctly excluded from the analysis in the study.

### Recruitment

**Figure 1:**
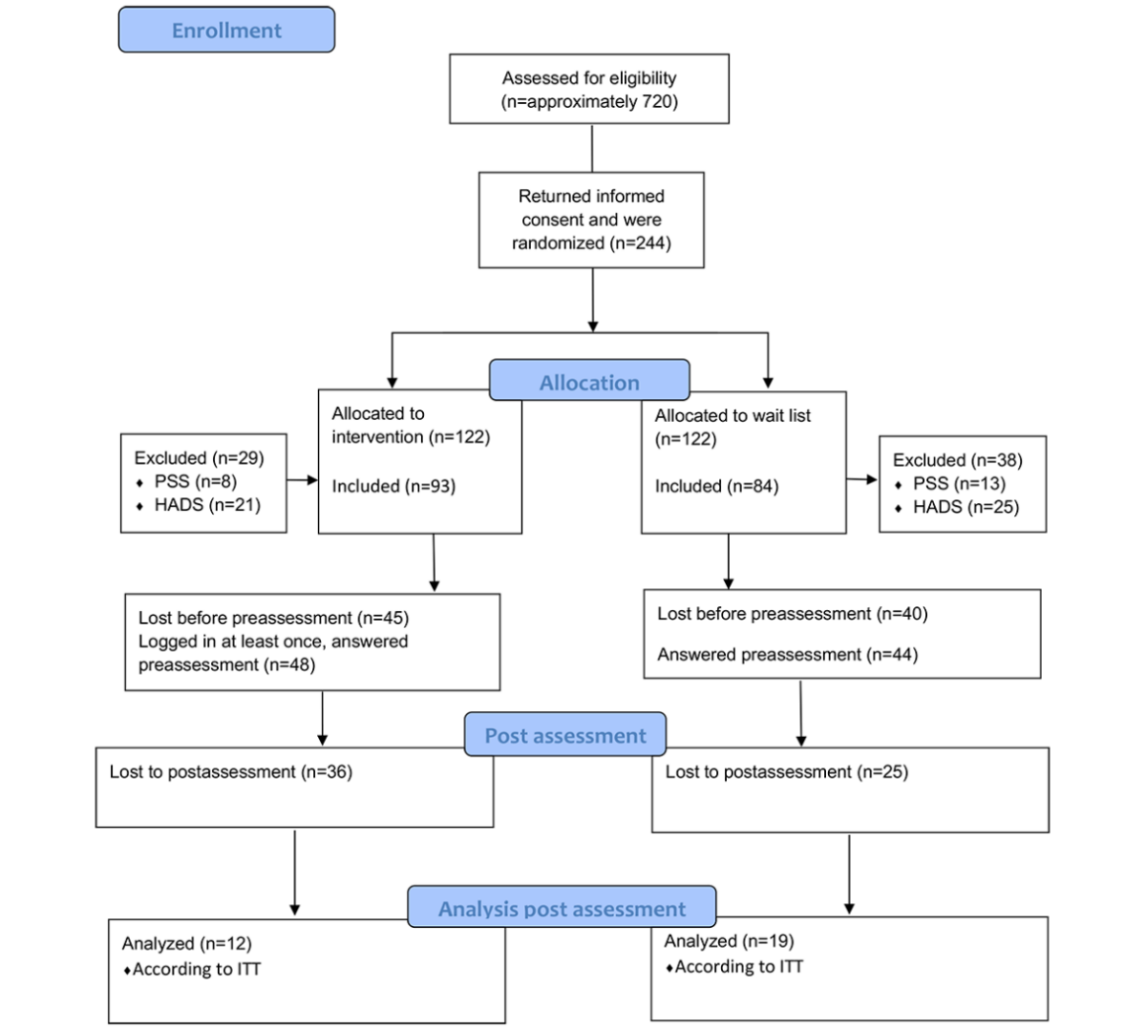
Flowchart of enrollment, randomization, and exclusion. HADS: Hospital Anxiety and Depression Scale; ITT: intention-to-treat; PSS: Perceived Stress Scale.

Participants were recruited by the first author visiting different work sites and workplace meetings at some of the largest work sites in the region. Employers with a larger number of staff required fewer contacts with executives and gatekeepers. All participants had access to a computer or tablet and internet connection, and most of them both at work and at home. The first author visited 28 different work sites, some of them twice, representing the private sector, municipality, and county council. Multimedia Appendix 2 shows the demographic characteristics of the participants. Eligible persons were informed both verbally and by an information letter about the study and inclusion and exclusion criteria. The inclusion criteria were a perceived stress score, measured with the PSS-14 [21], of 17 or higher [20]; being employed; being aged 18 to 65 years; able to speak and understand the Swedish language; and consenting to participate in the study. Exclusion criteria were being currently on sick leave or scoring 11 or more on either of the subscales of the HADS [22]. Persons who perceived themselves as stressed were encouraged to sign up for the study, return the signed informed consent form, and provide an email address for further communication. The enrollment and flow of participants are shown in Figure 1. All correspondence with the participants was through email with the first author. Recruitment for this study started during autumn 2016. The participants had access to MSC from December 2016 to May 2017. Follow-up measures were conducted on an ongoing basis.

### Randomization

To have persons from the same work site in both groups (intervention and wait-list groups), randomization was conducted by randomizing the participants from each work site to either the intervention or wait-list group in blocks of 6 persons. We chose blocks of 6 persons to be more certain that we would have an equal number of participants in both groups; we also included participants from work sites where few persons consented to participate. Randomization was conducted using a web-based randomizer [23] with a set of equal figures of ones and twos in a 6-number set, meaning that each set contained 3 ones (randomized to the intervention group) and 3 twos (randomized to the wait-list group). The first author performed the randomization procedure. See Multimedia Appendix 2 for demographic data, descriptive statistics for primary outcome measures, and secondary outcomes at baseline for all participants completing the first assessment.

### Intervention

The intervention group received access to the web-based program for self-management of stress, MSC [17,18], after a baseline assessment. MSC tailors to each user in 2 main ways: by recommending different stress management strategies depending on stress-related problems experienced by each unique user (using a functional behavioral analysis) and by assessing the stage of change [24,25]. The web application is fully automated and does not provide the user with any contact with a therapist or coach. Information in the program is delivered as text, film, and audio recordings. Feedback is provided using tables and charts of how stress levels and stress-related problems and symptoms are changed during the course of the program. The theoretical framework for the program includes SCT [26], Theory of Reasoned Action, Theory of Planned Behavior [27], Transactional Theory of Stress and Coping [28], and the Transtheoretical Model and Stages of Change [29]. SCT is the overarching theory of MSC and links the individual, behavior, and environment in a reciprocal manner. Furthermore, it is the basis for the psychoeducational module and the functional behavioral analysis where the user is educated about how the individual’s resources and environmental factors and behavior interact. MSC is also designed to support self-efficacy, a central concept in SCT, for coping with stressful situations. In Theory of Reasoned Action and Theory of Planned Behavior, identifying key behavioral beliefs and controlling these beliefs as determinants of behavioral intention is central [27]. This is integrated in MSC by components designed to increase perceived control over new behaviors. All modules, including stress management strategies, start with a film in which behavioral beliefs are addressed. Finally, the Transactional Theory of Stress and Coping includes the central concepts of primary and secondary appraisal and coping and influenced both the design of MSC as a program aiming at affecting both primary and secondary appraisal as well as coping and has influenced both the information throughout the program as well as the included stress management strategies.

Feedback in MSC is both ipsative and normative. Tailoring according to stages of change as well as recommended stress management strategies can be seen as a type of normative feedback. Normative feedback is also provided depending on the number of log-ins and also regarding examples the users receive on how to solve some of the assignments related to specific stress management strategies after completing them. The feedback the users of MSC receive after completion of each stress management strategy module is ipsative, and the users can follow their changes in stress-related symptoms in a table.

Screening is conducted when the users log in for the first time. The users are screened for stress levels using the PSS-14 [21,30] and for depression and anxiety with the HADS [22,31]. Stress scores <17 on the PSS-14 or ≥11 on one or both subscales of the HADS deny the user access to the program. To reach the intended persons, a cutoff score is used so that the users will reach a minimum stress level. The cutoff score on the PSS-14 was based on a study in which a stressed sample was divided into 2 groups of lower and higher stress levels [20]. The cutoff score of 17 in this study was based on the mean PSS-14 of the lower stress group minus one SD. This cutoff score was considered acceptable in a previous interview study [32].

**Figure 2:**
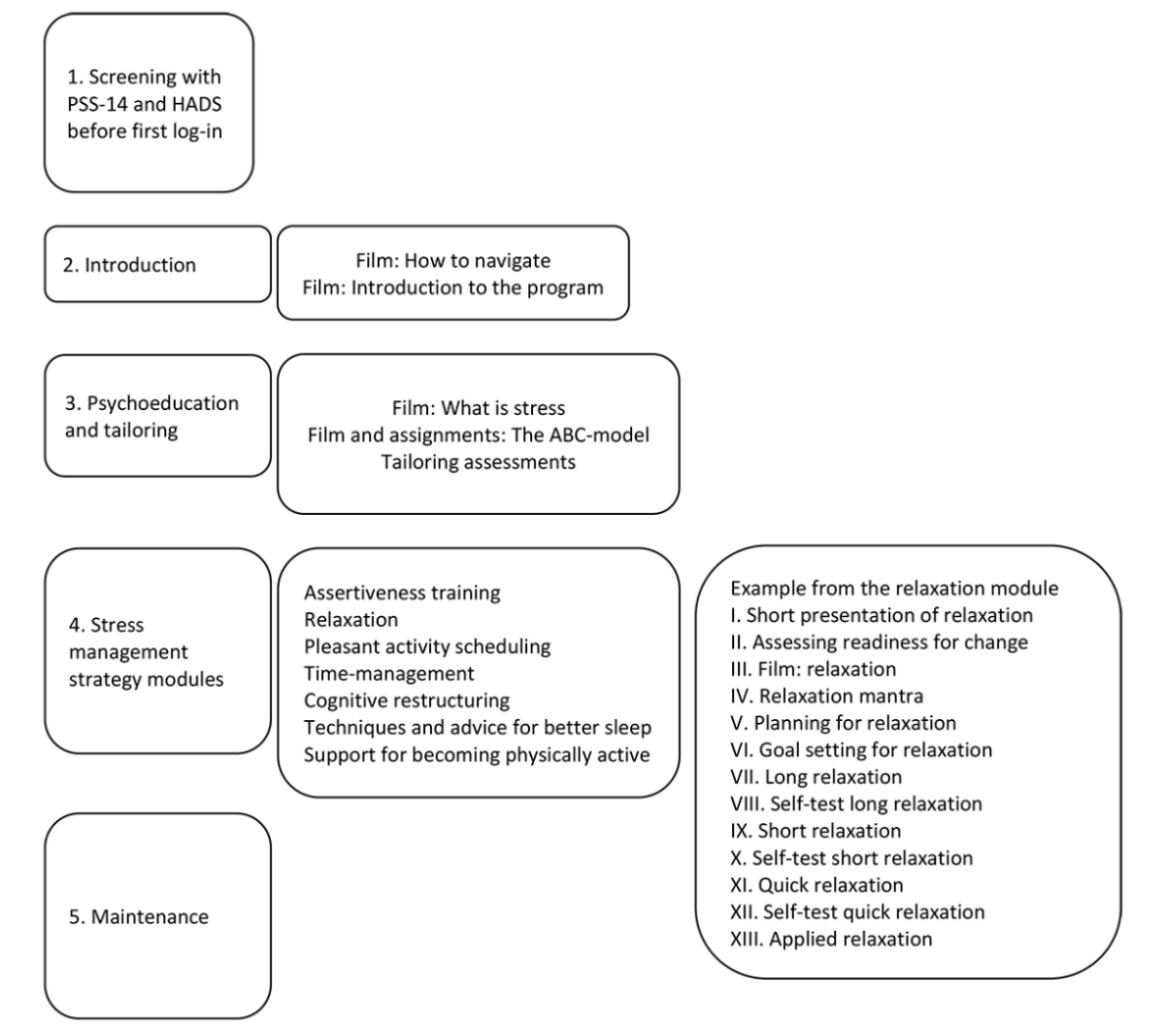
Overview of the flow through the program, content of each step and with a more detailed example from the relaxation module. ABC-model: Antecedent-Behavior-Consequence model; HADS: Hospital Anxiety and Depression Scale; PSS-14: Perceived Stress Scale, 14-item.

The MSC consists of 12 modules. All users must go through the first 2 modules: introduction to the program (including navigation and origin of the program) and psychoeducation (with information on what stress is, symptoms of stress, and how to lower stress). In the psychoeducation module, the specific needs of users for stress management were assessed. These individual needs are the basis for tailoring. The psychoeducation module prompts the user to conduct a functional behavioral analysis using an Antecedent-Behavior-Consequence model. An ambivalence module appears for those who are not ready to start a recommended stress management module in the program. There are 7 modules for stress management strategies (assertiveness training, relaxation, pleasant activity scheduling, time management, cognitive restructuring, techniques and advice for better sleep, and support for becoming physically active) and one module for maintenance of behavior. Users are recommended one stress management strategy at a minimum or all are recommended if relevant according to the tailoring. Most assignments in the modules are designed for working within 1 week, although the users are free to choose to work faster or stay with an assignment longer. See Figure 2 for an example from the relaxation module. In the final module, techniques for supporting maintenance and planning for future stress management training are provided. Techniques to support behavior change are integrated into every module. Prompting intention to change, self-monitoring, goal setting, re-evaluation of goals, and feedback on performance are the central techniques in the program [17,18,33]. See Multimedia Appendix 3 for the screen capture of the MSC. This intervention has been described elsewhere [17]. See Figure 2 for an exemplary run-through.

### Procedure

The participants received user names, passwords, and links to the questionnaires and the MSC at a preferred email address provided in the consent form. Only study participants had access to the program, and only as study participants, the program could be accessed. All questionnaires were converted to a web-based format and looked as similar as possible to the original version. The questionnaires were connected to the user names for the web application. All users had personal log-in, not connected to their name or work site, connected to the email provided. Most participants chose their work-connected email addresses. Information on security using the platform was provided, and participants were told that all communication to and from the web application was encrypted using the Secure Sockets Layer protocol. In addition, IP addresses from multiple failed log-ins were temporally banned. Backup procedures were performed regularly to avoid the loss of user data. The study participants were informed about the security of web-based applications.

### Outcome Measures

#### Primary Measures

The primary dependent measure was the Swedish version of the PSS-14 [21,30]. The PSS-14 assesses the frequency of stress-related thoughts and feelings using 14 items. Responses are given on a 5-point scale, ranging from *never* to *very often*. In 7 of the items, the response *very often* indicates high stress, whereas in the other half, the responses are reversed. Responses are summed to a total score ranging from 0 to 56, with high scores indicating high stress. PSS-14 was used both at baseline and postintervention assessments. The Swedish version [34] and the original English version [21] of the PSS-14 have shown good psychometric attributes. The Cronbach α was .75 at baseline assessment in this study.

#### Secondary Measures

The 26-item Coping Self-Efficacy Scale measures one’s perceived self-efficacy with coping behaviors when faced with challenges in life [35]. This scale has demonstrated good validity and reliability [35]. The items concern beliefs in performing behaviors important to adaptive coping scored on an 11-point scale, where 0 means *cannot do at all* and 10 means *being certain that one can do*. Items are summed to obtain a total score between 0 and 260. High scores indicate high self-efficacy in coping with stress. The Coping Self-Efficacy Scale was used both at baseline and postintervention assessments.

The General Nordic Questionnaire for Psychological and Social Factors at Work (QPS) measures psychological and social factors at work [36] and has shown good validity and reliability [37]. In this study, the short form, QPS Nordic 34+, was used [36]. It contains 37 items divided into 23 subscales and single items. All items are scored on a 5-point Likert scale. The mean of each subscale is calculated. The QPS Nordic 34+ questionnaire was used for both the baseline and postintervention assessments.

The shortened version of the Utrecht Work Engagement Scale [38] is a 9-item scale with 3 subscales measuring a person’s engagement in his or her work. All items are scored on a seven-point Likert scale (0-6). The Utrecht Work Engagement Scale yields 3 subscales (*vigor*, *dedication*, and *absorption*) and a total score. The mean for each subscale and the mean of the total scale are calculated. Each subscale consisted of 3 items. High scores indicate high work engagement, which is negatively related to burnout. This questionnaire was used at both baseline and postintervention assessments. The scale has shown acceptable psychometric properties [38], and the Swedish version has shown good reliability [39].

The situational version of the Brief Coping Orientation to Problems Experienced Inventory (COPE) [40] is a 28-item scale with a four-point response scale that measures coping strategies in stressful situations. It has shown good reliability [41] and validity [42,43]. Each item ranges from *never* to *very often* and measures 14 different coping strategies. A confirmatory factor analysis [44] led to the conversion of the original 14 subscales into 6 subscales (used in this study): self-distraction (scoring 2-8), problem-focused coping (scoring 4-16), avoidant coping (scoring 6-24), socially supported coping (scoring 6-24), emotion-focused coping (scoring 8-32), and self-blame (scoring 2-8) [44]. In addition, two 4-item emotional approach coping scales were embedded in the Brief COPE [45]. The items for each subscale are summed, except for the 2 last subscales about coping through emotional processing and emotional expression, where the means of the items are calculated. The range of scores on the last 2 subscales ranged from 1 to 4. This questionnaire was used at both baseline and postintervention assessments.

The Motivation for Change Questionnaire (MCQ) [46-48] measures motivation for change in life and work situations. The MCQ showed good reliability [46] and validity [47] in the development samples. The MCQ contains 48 items forming 7 subscales related to life situations and 6 subscales related to work situations. The subscales related to life situations are social support in life, control in life, mastery in life, challenges in life, values, self-efficacy, and self-confidence. The subscales related to work situations are coworker support, supervisory support, challenges in work, job control, interactions, and goals. Owing to technical errors, the subscale interaction was omitted in this study. Responses are given on a 4-point scale, ranging from *never* to *very often*. The scoring is reversed for 5 items. The median of each subscale is calculated. High scores indicate high motivation [48]. The MCQ was used to study whether motivation to change could predict adherence to MSC and was used at baseline assessment.

Internal consistency was calculated for the measures at the preassessment, both for total scales and subscales for the secondary outcomes, with the Cronbach α or Spearman-Brown coefficient as applicable. The reliability was 0.7 or higher for all total scales and subscales save for 3 subscales of the Brief COPE (self-distraction: 0.4; avoidant coping: 0.5; and emotion-focused coping: 0.6), 7 of the subscales of the MCQ (control in life: 0.3; mastery in life: 0.6; challenges in life: 0.4; values: 0.4; self-efficacy: 0.6; job control: 0.3; and goals: 0.5), and 5 of the subscales of the QPS (positive challenges at work: 0.6; control over decisions: 0.2; innovative climate: 0.6; inequality: 0.4; and work satisfaction: 0.6).

### Procedure for Data Collection

The exclusion and inclusion criteria were measured using the PSS-14 and HADS after randomization. Demographic data were collected together with baseline assessments after randomization. This procedure was chosen because the PSS-14 and HADS were built into the screening section after opening the program for the first time. The postintervention assessment was sent out 4 months after the initial log-in. Reminders were sent out 2 and 4 weeks after each time point, including the baseline. The wait-list group received the assessments at the corresponding time points as the intervention group from the same work site. All data were collected in a web-based format.

### Wait List

The persons in the wait-list group received questionnaires on 2 occasions. The first occasion coincided with the preintervention assessment for the intervention group, and the second occasion was 2 months after the first assessment. The 2-month follow-up was decided, as this time point was estimated to be the shortest time frame for completing the intervention in the intervention group. After the second assessment, the wait-list group received access to MSC.

### Statistical Analysis

Analyses were performed using SPSS 24 [49]. The significance level was set at *P*<.05. Student *t* tests (2-tailed) were used for differences between and within intervention and wait-list groups on the PSS-14 and demographic data. Chi-square and Fisher exact tests were used to detect differences between the 2 groups on categorical demographic data. Demographic data were presented using descriptive statistics. Comparisons of demographic data and outcome measures were conducted between dropouts and completers of the 2 assessments in the intervention and wait-list groups using the Student *t* test, chi-square test, or Fisher exact test. Effect sizes were calculated by adjusting the calculation using the pooled SD (Cohen *d*) [50].

For secondary outcome measures, Wilcoxon signed rank tests were used to calculate within-group changes, and Mann-Whitney *U* tests were used for between-group differences at baseline and for between-group changes at the postintervention assessment. Mann-Whitney *U* tests were also used for dropout analysis for secondary outcomes for differences between completers and dropouts.

The primary and secondary outcome analyses were conducted according to the intention-to-treat. The data for the primary outcome, PSS-14, were normally distributed without any outliers.

Participants who completed 2 rounds of assessment were defined as completers. Dropouts were defined as participants who completed the first assessment only.

### Compliance With Ethical Standards

All procedures performed in studies involving human participants were conducted in accordance with the ethical standards of the institutional and national research committee and with the 1964 Helsinki declaration and its later amendments or comparable ethical standards. Informed consent was obtained from all participants included in the study. The ethical application of Dnr 2015/555 was approved on January 20, 2016, by the Regional Ethical Review Board in Uppsala County, Sweden. An amendment application was approved for this study on December 16, 2016.

## Results

### Effects of MSC

For the primary outcome, the PSS-14, there were no significant differences between the intervention (mean 3.1, SD 7.66) and the wait-list group (mean 1.42, SD 6.16) in mean change from baseline to postintervention assessment. A small effect size was detected for the between-group mean change (Cohen *d*=0.25). There were no significant within-group differences in either the intervention group from preassessment (mean 24.25, SD 4.67) to postassessment (mean 21.17, SD 10.54) or in the wait-list group from preassessment (mean 24.26, SD 6.26) to postassessment (mean 22.84, SD 5.24) on PSS-14. Small within-group effect sizes were observed (Cohen *d*=0.38) in the intervention group and the wait-list group (Cohen *d*=0.25).

There were differences between the intervention and wait-list groups in two secondary outcomes: the subscale of coping through emotional processing (*Z*=−2.3; *P*=.02) from the Brief COPE and predictability (*U*=46.5; *P*=.03) from the QPS.

There were significant within-group changes for the completers in the intervention group on 2 secondary outcome measures. The subscale of self-blame from the Brief COPE was significantly lower in the intervention group at the postintervention assessment (*Z*=2.06; *P*=.04). In addition, in the intervention group, there were significant within-group differences in the 2 subscales from the QPS, showing higher role conflicts (*Z*=2.06; *P*=.04) and lower stress (*Z*=2.43; *P*=.02) at the postintervention assessment. Role conflict was reported to be significantly higher in the wait-list group from pre- to postintervention assessment (*Z*=2.39; *P*=.02), and there were more problematic situations with social interaction (*Z*=−2.12; *P*=.03). Multimedia Appendix 4 shows the medians and ranges of the pre- and postintervention scores for completers in the intervention and wait-list groups.

### Dropout Analysis

There were no differences in demographic data between the completers and dropouts in the intervention group. See Multimedia Appendix 5 for medians and ranges of the preintervention assessment for dropouts in the intervention group and dropouts in the wait-list group. In the wait-list group, there was a significant difference between completers and dropouts regarding the sector in which the participants were employed. Persons in the private sector had the highest dropout rate (100%).

There was a significant difference in perceived stress, the PSS-14, between the completers (mean 24.25, SD 4.67) and dropouts (mean 27.69, SD 5.25) in the intervention group (*t*=−2.0; *P*=.049). Self-efficacy, measured with a subscale of the MCQ, was significantly higher in the intervention group than the dropouts in the intervention group (*U*=110.5; *P*=.02). For the QPS, support from colleagues was significantly higher in the intervention group than for dropouts in the intervention group (*U*=97.5; *P*=.02), and the completers had a better perceived social climate (*U*=108.5; *P*=.049) and teamwork (*U*=68.5; *P*=.002). On the Brief COPE, the intervention group reported significantly higher use of coping through emotional processing (*U*=77.5; *P*=.01) compared with the dropouts.

In the wait-list group, the subscales of vigor (*U*=65; *P*<.001), dedication (*U*=95; *P*=.01), and absorption (*U*=74.5; *P*<.001) and the total score on the Utrecht Work Engagement Scale (*U*=67; *P*<.001) were all significantly higher in completers than the dropouts in the wait-list group. On the QPS, role clarity was significantly higher in completers than the dropouts in the wait-list group (*U*=98; *P*=.03), as was predictability (*U*=87; *P*=.01), experience of mastery (*U*=99.5; *P*=.02), social climate (*U*=100; *P*=.03), innovative climate (*U*=98; *P*=.02), teamwork (*U*=78; *P*=.004), and work satisfaction (*U*=85.5; *P*=.01). There was also significantly lower role conflict in completers than the dropouts in the wait-list group (*U*=31; *P*<.001).

## Discussion

### Principal Findings

In this study, a web-based self-management program for stress management named MSC was evaluated for its effect on users’ perceived stress, coping self-efficacy, work engagement, coping strategies, and psychosocial factors at work compared with a wait-list group. The main results showed that there were no significant differences in the outcome measures between the wait-list group and the intervention group in the primary outcome (perceived stress). However, a small effect size was found for perceived stress both between the intervention and wait-list groups and within the intervention group as well as within the wait-list group.

### Comparison With Prior Work

A meta-analysis of stress management interventions in occupational settings showed that the interventions, on average, had a medium to large effect on stress [51]. However, most of the interventions in the meta-analysis were face-to-face or relaxation interventions using audio tapes. For web-based stress management, one study showed a large effect size [16], but that intervention included feedback from an e-coach, a person giving written feedback and sending reminders, thereby placing a greater demand on resources than MSC. Few resources are necessary to administer and deliver MSC because it is a fully automated program. A program demanding few resources with a small effect size could be considered important and could still be worth investigating with MSC in a larger study. A stress management program that demands fewer resources focused on stress-preventive actions in persons not on sick leave is valuable for preventing stress-related ill health. A score of 25 or higher on the PSS-14 has been used as a cutoff for high stress levels [20]. In this study, the median for perceived stress in the intervention group was 24 at preassessment and 19.5 at postassessment. In a previous population study, the average stress level was 17 in the PSS-14 [30]. Thus, our participants decreased their stress level to near the population level of 17, as seen in an earlier study [30], and our study participants may have reached their potential for change. This factor may have contributed to the nonsignificant differences between groups. Thus, difficulties in finding significant changes were expected because this application was developed for health promotion, and the study participants were not expected to have very high levels of stress at baseline.

The wait-list group reported higher role conflict postintervention assessments. To have yet another task to complete, such as an extensive battery of questionnaires, additional persons to satisfy (the researchers) could lead to feelings of conflicting roles. Nevertheless, there was a small effect size on perceived stress in the wait-list group over time. Effects in the wait-list group have also been reported in other studies [16]. This effect could depend on how a person in an ongoing study becomes aware of, in this case, his or her stress-related problems and automatically starts to handle them and changes their behavior. Filling in the questionnaires could be considered self-monitoring, which is known as a strong technique for creating behavior change [33]. However, the effect in the wait-list group could also depend on contamination between the intervention group and the wait-list group because the participants from the same workplace were included in both groups.

It could be considered controversial to launch a program for individual stress management because work-related stress is often described as deriving from organizational features [1,52] and demands [4] at work. Putting a lot of focus on individual responsibility to, on his or her own, handle stress that might have been caused by work conditions could make the individuals feel that the stress is their responsibility. Nevertheless, the fact that self-blame was significantly lower at postintervention assessment in the intervention group indicates that the goal of educating the participants on stress, the causes of stress, and the role that both environmental and individual factors have in stress management was successful and even contributed to lower self-blame.

Only 25% (12/48) of the intervention group completed the postintervention assessment. Adherence in this study was thus low compared with that in earlier studies [14]. The low adherence could be associated with the technical issues a handful of early participants encountered, the high number of questionnaires used, and the program was perceived as extensive. The technical issues, which led to irritation among the early participants, may also have contributed to the higher dropout rate in the intervention group compared with the wait-list group. Although the feasibility of MSC was evaluated in our previous studies [18,32], and the MSC was further developed to be less extensive, it may still have been too extensive and perceived as too complicated for the participants to feel motivated to use it. The results from previous studies on MSC also influenced how the program was presented to potential study participants so the study participants would have realistic expectations about the program in terms of the time required to use it. As mentioned previously, access to a coach or therapist has been shown to support adherence [14,16], but it can also hinder the distribution of a web application because real-life support requires more resources than a fully automated program such as MSC. Providing an e-coach is resource demanding, and one study reported that the e-coach spent approximately 30 minutes for each person and module completion [16]. The fully automated nature of MSC may have contributed to the low study adherence, even if tailored but automated feedback was provided. Adherence to MSC may benefit from having some kind of e-coach giving individualized feedback and prompting the participants to adhere to the program and assignments. In MSC, a problem-solving model for identifying social support to identify persons who could, for example, be involved as a coach, was integrated into one of the final modules of the MSC to support better adherence to the recommended assignments. The problem-solving module may work earlier in the program. A weakness of this study was that use and adherence to assignments were not monitored and the dose the study participants received was not known. The choice not to monitor was due to the tailoring, that the study participants were free to choose parts other than the recommended and work at their own pace.

In future trials of MSC, the number of log-ins and use of the different functions in MSC should be more carefully monitored to understand how the users use MSC. In the administrations tool of MSC, the users were followed regarding how many times they logged in but not for how long they used the app, which means that they could have been logged in for several days without logging out. In future studies of MSC, the administration tool needs to have more detailed information and tracking possibilities regarding traffic and use (eg, time spent on each assignment) and the functions that were used the most.

The dropout analysis revealed some significant differences between completers and dropouts in both the intervention and wait-list groups. In the intervention group, completers had higher self-efficacy, higher support from colleagues, better social climate, and scored higher on teamwork, whereas dropouts had significantly higher stress at baseline. These differences may indicate that the completers perceived having resources, both external and internal, to work with their own stress management using MSC. Exposure to psychosocial risk factors at work, such as low coworker support, has been associated with stress-related health problems [53]. The findings in this study imply that those with the most to gain in preventing stress-related ill health dropped out to a greater extent. Thus, a stress management program such as MSC can never be seen as an absolute solution to work-related stress, and psychosocial risk factors for stress must also be handled at an organizational level [54]. A focus on heightening self-efficacy [55] has been associated with reduced absences from illness in coping-related interventions in an occupational setting [56,57]. Thus, self-efficacy could be considered an important resource for maintaining health when exposed to stress. MSC is developed based on evidence to strengthen self-efficacy [58], but it seems important to find ways for those with low self-efficacy to engage in the intervention. Completers also reported higher use of coping through emotional processing, indicating that they may have been more prone to take time to reflect on their own situation. Coping through emotional processing has been identified as an adaptive coping strategy [59]. Indications that MSC evokes self-reflective processes were also found in our previous study evaluating the feasibility of an earlier version of MSC [32].

The dropouts from the wait-list group could be considered similar to the dropouts from the intervention group in that they reported more exposure to psychosocial situations at work. They reported a lower innovative and social climate at work, scored lower on teamwork, reported lower work satisfaction, role clarity, predictability, and lower experience of mastery. This similarity could guide researchers in environmental circumstances at work that hinder participation when perceiving oneself as stressed but might also depend on how people with fewer internal and external resources do not prioritize completing the multiple questionnaires, which was an issue identified in an earlier study [32].

The amount of time required to complete the study questionnaires negatively affected adherence to the program. In this study, several closely related domains of stress and factors at work were studied, and data were collected with a relatively high number of items. However, work-related stress is complex; the causes of developing stress-related ill health are multifactorial, and the experience of the consequences of stress is varied. The rationale for using multiple questionnaires was to capture the multifactorial aspects of stress in a work-related context. This choice was also a relevant choice in light of the difficulties in operationalizing work-related stress. The second round of measurements may have been a barrier for adherence, and more persons might have used the program than those who answered the second round of measures. In future trials, the possibility of asking for reasons for dropouts should be investigated. Nevertheless, this is an ethical dilemma that needs to be further investigated because the study participants were informed that they could drop out from the study at any point without giving reasons.

The behavior change model for internet interventions describes the factors affecting behavior change in web-based interventions. According to the model, adherence is affected by the characteristics of the user and the program, degree of support, and environmental factors [60]. All possible characteristics and needs of the users intended for MSC may not have been considered when developing the MSC. For example, the program needs to support users in prioritizing self-management. To handle difficulties in prioritizing self-management, there is a time management module in MSC [17]. This module could have been integrated partly in the psychoeducation module to help study participants prioritize their own stress-related problems at an early stage. It is also possible that MSC needs to be simpler in both content and technology. Furthermore, reminders have been shown to be an important characteristic of a program that facilitates adherence [61], but few web-based interventions use them [14], which could depend on the related costs for an SMS or technical difficulties such as deciding the best time to send reminders. A function for sending out reminders should be further developed to facilitate adherence to MSC.

One of the strengths of this study was the RCT design, even if a weakness was the lack of blinding in this study. In an RCT with a proper randomization procedure, it is possible to control for factors that might influence the results, in addition to the intervention. In addition, earlier research has shown that it is a strength [62] to build programs such as MSC on theory. The content of the program and causalities between its concepts can be motivated, validated, and understood by theories. In addition, integrating several scientific fields in program development could contribute to a more holistic program in terms of content and presentation.

The randomization procedure in blocks of 6 persons was chosen to increase the chance for persons from the same work sites to be randomized to both groups. This randomization was desired to minimize the risk of other work-site–related incidents to bias the results. This procedure can be considered a risk factor for contamination [63]. Having persons from the same work site in both groups could have contributed to the small positive effect size in the wait-list group because of contamination. However, baseline data showed that randomization was successful. The randomization was conducted before screening for depression and anxiety as well as before they completed the baseline. This might have had a negative effect on study attrition and could be a limitation of this study. This strategy was chosen because the application (MSC) was developed to screen for depression and anxiety, as well as for perceived stress levels, and was designed to be as close as possible to how MSC is planned to be distributed in the future, except for the integrated measurements for primary and secondary outcomes for the study purpose.

At baseline, no significant difference between the intervention group and wait-list groups was detected regarding the work sector (private, municipality, and county council; Multimedia Appendix 2).

In addition, the small sample size is a limitation that could have caused the nonsignificant effects, thus increasing the risk of type II error. The sample size was insufficient to provide power for this study. Although one additional work site was included after the start of the study, the included work sites were depleted. Although ad hoc, the expected dropout rate has been estimated to be 50% instead of 20%, which is in line with the average dropout from web-based applications for behavior change and self-management of different health-related conditions [14]. Nevertheless, a focus on features that facilitate adherence was hypothesized to increase adherence to MSC compared with earlier web-based stress management programs, which was not enough to facilitate adherence to MSC. Nonetheless, nonsignificant results in a small sample can still produce a relevant effect size. Thus, MSC should be further studied for its effect on perceived stress, but with a focus on support adherence in the indented group.

Gamification has been used in several studies to improve mental health and well-being [64]. In a systematic review, the argument for gamification in the included papers was to promote engagement and enhance an intervention’s intended effect [64]. The authors of the systematic review also identified common gamification elements such as levels or progress feedback, points or scoring, rewards or prizes, narrative or theme, personalization, customization, artificial assistance, unlockable content, social cooperation, exploratory or open-world approach, artificial challenge, and randomness [64]. Even if MSC have some of these functions, for example, levels or progress feedback, personalization, and unlockable content, it may be useful in subsequent iterations of the intervention to have more engaging mechanisms built in the application. Gamification may have a positive effect on how participants experience the intervention by increasing cognitive engagement and the combination of cognitive and affective engagement [65].

There was a significant difference in dropouts between the different sectors. This difference could be associated with the postintervention assessment and reminders that were sent out during a summer vacation period to the persons employed in the private sector. The timing of starting an intervention and planning the postintervention assessment must be considered in future studies.

Approximately 30% (244/720) of the eligible participants provided informed consent. This percentage includes both included and excluded persons. On the basis of this percentage, issues with selection bias must be considered. Consequently, the external validity of the results is limited to persons with perceived stress who are willing to find solutions on an individual level. Participants in the intervention group who reported higher general self-efficacy at baseline were more likely to complete the 2 rounds of assessments. This finding could indicate that the program suits persons who believe they have resources to handle stress at an individual level. In addition, the participants in this study were mostly women, which must be considered when generalizing the results.

When analyzing with intention-to-treat, there is a risk of type II errors [66], but intention-to-treat was chosen instead of per protocol because we did not study the extent to which participants took part in and adhered to the assignments.

The procedure for introducing and using MSC in this study was designed to, as far as possible, resemble how it would be done if MSC was a commercial product except the baseline and postassessment.

### Conclusions

It is still uncertain what effect MSC may have on perceived stress, but the effect size regarding the primary outcome indicates that it could be worthwhile to develop and evaluate MSC further. The result must be interpreted with caution because of the high dropout rate, which may have biased the results. As the focus of this study was to prevent stress levels that could lead to sick leave, the participants were not expected to have very high initial stress levels. Consequently, the participants’ potential to decrease their stress scores might have been rather limited, which probably contributed to the small effect size and nonsignificant results. Moreover, the power in this study was too low to ensure significant differences. MSC could, after further development and evaluation, be an alternative or complement to face-to-face interventions, as stress management could be valuable for decreasing stress on a large scale in the workforce, but further studies that focus on facilitating adherence are required. In addition, further studies should focus on determining the effective elements of the program to condense the program. Condensing the program may, in turn, contribute to higher adherence rates. Furthermore, the length of the test battery in the study may have been a heavy burden to the participants, and the study may have reduced the length of the tests and thus might have contributed to the low adherence. Finally, the technical issues encountered may have been prevented by more extensive beta testing with the target group.

## Appendix

Multimedia Appendix 1 CONSORT-EHEALTH (Consolidated Standards of Reporting Trials of Electronic and Mobile Health Applications and Online Telehealth) checklist.

Multimedia Appendix 2 Demographic data, descriptive statistics for primary outcome measures, and secondary outcomes at baseline for all participants completing the first assessment.

Multimedia Appendix 3 Screen capture video of My Stress Control (MSC) showing log-in, screening for depression, anxiety and stress levels, film regarding navigation of MSC, introduction to MSC, Antecedent-Behavior-Consequence model, and time management module.

Multimedia Appendix 4 Pre- and postintervention assessment for completers in the intervention group and completers in the wait-list group.

Multimedia Appendix 5 Medians and ranges in the pre- and postintervention assessments for dropouts in the intervention group and dropouts in the wait-list group.

## Abbreviations

CONSORT-EHEALTH: Consolidated Standards of Reporting Trials of Electronic and Mobile Health Applications and Online Telehealth
COPE: Coping Orientation to Problems Experienced Inventory
HADS: Hospital Anxiety and Depression Scale
MCQ: Motivation for Change Questionnaire
MSC: My Stress Control
PSS-14: Perceived Stress Scale, 14-item
QPS: General Nordic Questionnaire for Psychological and Social Factors at Work
RCT: randomized controlled trial
SCT: Social Cognitive Theory
