# Supplementary material for: Evaluation of a Web-Based Stress Management Program for Persons Experiencing Work-Related Stress in Sweden (My Stress Control): Randomized Controlled Trial
Source: JMIR Ment Health. 2021 Dec 9;8(12):e17314. doi: 10.2196/17314 (PMC8704112; doi:10.2196/17314)
Supplement: Multimedia Appendix 2 [file mental_v8i12e17314_app2.pdf]

Multimedia Appendix 2. Demographic data, descriptive statistics for primary outcome measures, and secondary outcomes at baseline for all participants completing the first assessment.

Characteristics		Intervention (n=48)	Wait-list (n=44)
Age (years), mean (SD)		45.9 (10.5)	40.5 (10.2)
Sex, female, n (%)		42 (88%)	39 (89%)
Percent working of full-time, mean (SD)		98% (19.1)	97% (7.7)
**Marital status, n (%)**			
	Living alone	6 (13)	11 (25)
	Living with partner	15 (31)	12 (27)
	Married	27 (56)	21 (48)
**Education, n (%)**			
	Compulsory school	1 (2)	0
	Upper secondary school	8 (17)	12 (27)
	Bachelor degree	34 (71)	26 (59)
	Master degree	5 (10)	6 (14)
**Work sector, n (%)**			
	Private	5 (10)	8 (18)
	County council	19 (40)	21 (48)
	Municipality	24 (50)	15 (34)
**Employment, n (%)**			
	Permanent	46 (96)	42 (96)

	Temporary	1 (2)	2 (5)
	By hours	1 (2)	0
Chronic illness, n (%)		9 (19)	8 (18)
Reports sick leave last year, n (%)		10 (21)	5 (11)
Sick leave during last year, n, mean (SD)		n=6, 66.7 (51.6)	n=9, 55.6 (52.7)
Sick leave (weeks), n, mean (SD)		n=9, 5.6 (5.7)	n=9, 1.6 (2.4)
Perceived Stress, mean (SD)		26.83 (5.21)	26.23 (5.9)
Coping self-efficacy scale, median (IQR, 25th percentile:75th percentile)		n=45	n=42
		148 (121.5:166)	143.5 (117.3:165.5)
**Utrecht Work Engagement Scale, median (IQR, 25th percentile:75th percentile)**		n=44	n=39
		4.2 (3.6:4.7)	4.3 (3.6:5)
	Vigor	4 (3.3:4.6)	4 (3.3:5)
	Dedication	4.3 (3.7:5)	4.7 (4:5.3)
	Absorption	4 (3.3:4.7)	4 (3.7:5)
**Brief Coping Orientation to Problems Experienced Inventory, median (IQR, 25th percentile:75th percentile)**		n=41	n=37
	Self-distraction	5 (4:6)	5 (3.5:6)
	Problem-focused coping	12 (11:12)	12 (10:13)
	Avoidant coping	9 (7:10)	9 (8:10)

	Socially supported coping	15 (13:18)	15 (13:17)
	Emotion-focused coping	19 (17:21)	18 (15.5:20.5)
	Self-blame	5 (4:6)	5 (4:6)
	Coping through emotional processing	2.5 (2:3)	2.5 (2:2.8)
	Coping through emotional expression	2.3 (1.5:2.8)	2.3 (2:2.8)
**The short version of the General Nordic Questionnaire for Psychological and Social Factors at Work, median (IQR, 25th percentile:75th percentile)**		n=43	n=37
	Quantitative demands	3.5 (3:4)	4 (3.3:4.3)
	Demands on learning	2.5 (1.5:3)	2.5 (1.5:3)
	Role clarity	4 (3.5:4)	4 (3:5)
	Role conflicts (single item)	2 (2:3)	3 (1:3)
	Positive challenges at work	4 (3.5:4.5)	4.5 (4:4.5)
	Control over decisions	3 (2.5:3)	3 (2.5:3)
	Control over working pace	3.5 (3:4)	3.5 (2.3:4.5)

	Predictability over next month (single item)	4 (2:4)	4 (2:5)
	Predictability (single item)	3 (2:4)	3 (2:4)
	Experience of mastery (single item)	4 (3:4)	4 (3:4)
	Support from employer	4 (3:4)	3.5 (3:4)
	Support from colleagues (single item)	4 (4:5)	4 (4:5)
	Support from friends and family (single item)	4 (3:4)	4 (3:4)
	Social interaction (single item)	3 (2:3)	3 (2:4)
	Encouraging leadership	3.5 (3:4)	3.5 (2.5:4)
	Social climate	4 (3.5:4)	4 (3.5:4.5)
	Innovative climate	4 (3.5:4)	3.5 (3:4)
	Inequality	1 (1:2)	1 (1:2)
	Personnel targets	3 (2.5:3.5)	2.5 (1.8:3.5)

	Organizational culture and climate (single item)	3 (3:4)	3 (3:4)
	Teamwork	4 (3.5:4.5)	4.5 (3.5:4.5)
	Work satisfaction	3.5 (2.5:4)	3.5 (2.8:4)
	Stress (single item)	3 (3:4)	4 (3:4)
Motivation to Change Questionnaire, median (IQR, 25th percentile:75th percentile)		n=43	n=42
	Social support in life	3 (3:3)	3 (2.5:3)
	Control in life	3 (3:3)	3 (3:3)
	Mastery in life	3 (3:3)	3 (3:3)
	Challenges in life	3 (3:3.5)	3 (3:3.5)
	Values	3 (3:3)	3 (2:3)
	Self-efficacy	3 (3:4)	3 (3:4)
	Self-confidence	3 (3:4)	3 (3:4)
	Coworker support	3 (3:3)	3 (3:4)
	Supervisory support	3 (2:3)	3 (2:3)
	Challenges in work	3.5 (3:3.5)	3.5 (3:4)
	Job control	3 (3:3)	3 (2:3)
	Goals	3 (3:3.5)	3.5 (3:3.5)
