# Supplementary material for: Evaluation of a Web-Based Stress Management Program for Persons Experiencing Work-Related Stress in Sweden (My Stress Control): Randomized Controlled Trial
Source: JMIR Ment Health. 2021 Dec 9;8(12):e17314. doi: 10.2196/17314 (PMC8704112; doi:10.2196/17314)
Supplement: Multimedia Appendix 4 [file mental_v8i12e17314_app4.pdf]

Variables (min-max)	Intervention group (completers)		Wait-list (completers)	
	Pre	Post	Pre	Post
	Median (IQR, 25th percentile:75th percentile)	Median (IQR, 25th percentile:75th percentile)	Median (IQR, 25th percentile:75th percentile)	Median (IQR, 25th percentile:75th percentile)
Perceived stress scale-14 (0-56)	n=12	n=12	n=19	n=19
	24 (20.5:28.3)	19.5 (15.8:25:8)	22 (20:28)	22 (19:27)
Coping self-efficacy scale (0-260)	n=12	n=11	n=19	n=18
	158.5 (111.3:179)	175 (119:197)	146 (119:171)	154 (134.8:175)
Utrecht Work Engagement Scale (0-6 for total/each subscale)	n=11	n=11	n=19	n=17
	4.3 (4.1:4.8)	4.7 (3.9:4.9)	4.7 (4.2:5.2)	4.9 (4.6:5.3)
Vigor	4.3 (4:4.7)	4.7 (3.3:5)	4.7 (4:5.3)	5 (4.7:5.2)
Dedication	4.7 (4:5)	4.7 (4:5)	5 (4.3:5.7)	5.3 (4.3:5.7)
Absorption	4.3 (4:5)	4.7 (4:5)	4.7 (4:5)	5 (4.3:5.3)
Brief COPE questionnaire	n=11	n=11	n=19	n=16
Self-distraction (2-8)	5 (4:6)	4 (3:5)	5 (4:6)	5 (4:5.8)

	Problem-focused coping (4-16)	11 (10:13)	11 (10:12)	12 (10:13)	11 (10:12.8)
	Avoidant coping (6-24)	9 (7:10)	7 (6:10)	9 (7:9)	8 (7:9.8)
	Socially supported coping (6-24)	15 (12:17)	14 (11:16)	15 (13:17)	14.5 (11:19)
	Emotional-focused coping (8-32)	19 (17:20)	18 (16:21)	19 (15:21)	(16.3:21.8)
	Self-blame (2-8)	5 (4:6)	4 (3:5)	4 (3:6)	4 (4:5.8)
	Coping through emotional processing (1-4)	3 (2.5:3.3)	3 (2:3)	2.5 (2:2.8)	2.8 (2.1:3)
	Coping through emotional expression (1-4)	2.5 (2:2.8)	2.5 (2.3:3)	2.3 (2:2.7)	2.3 (2:2.9)
QPS Nordic 34+ (1-5 for each subscale)		n=11	n=11	n=19	n=16
	Quantitative demands	4 (3:4)	4 (3:4.5)	3.5 (3:4.5)	(2.6:4)

	Demands on learning	2 (1.5:2.5)	2 (2:3)	2 (1:2.5)	1.8 (1.5:3)
	Role clarity	4 (3:4.5)	4 (3.5:5)	4 (4:5)	(3.6:4.9)
	Role conflicts (single item)	2 (1:2)	2 (2:4)	1 (1:2)	(1.3:3.8)
	Positive challenges at work	4 (4:4.5)	4.5 (4:4.5)	4.5 (4:5)	(4:5)
	Control over decisions	3 (2.5:3)	3 (2.5:3)	2.5 (2:3)	(2.6:3.5)
	Control over working pace	3 (3:3.5)	3.5 (3:3.5)	3.5 (3:4.5)	3 (2.1:4.4)
	Predictability over next month (single item)	4 (2:5)	3 (2:4)	4 (1:5)	(2:5)
	Predictability (single item)	3 (2:4)	4 (2:4)	3 (1:3)	2.5 (2:3)
	Experience of mastery (single item)	4 (4:4)	4 (4:5)	4 (4:5)	4 (4:5)
	Support from employer	3.5 (3.5:4.5)	4 (3:4)	4 (3:4.5)	3.8 (3.5:4.5)

	Support from colleagues (single item)	5 (4:5)	4 (4:5)	4 (4:5)	4 (4:5)
	Support from friends and family (single item)	4 (3:4)	4 (3:5)	4 (3:4)	4 (3:4)
	Social interaction (single item)	3 (2:3)	3 (2:4)	2 (2:4)	3 (2:3)
	Encouraging leadership	3.5 (3:4.5)	3.5 (3:4)	3.5 (2.5:4)	3.5 (3.1:4)
	Social climate	4 (4:4)	4 (3.5:4)	4 (4:4.5)	4.5 (3.6:4.9)
	Innovative climate	4 (3.5:4.5)	3.5 (3.5:4.5)	4 (3:4)	4 (3.5:4.5)
	Inequality	1 (1:2)	1 (1:2)	1 (1:2)	1.5 (1:2)
	Personnel targets	3 (2.5:4)	2.5 (2:4)	3 (2.5:3.5)	3 (2.5:3.5)
	Organizational culture and climate (single item)	4 (3:4)	3 (2:4)	4 (3:4)	4 (3:4)
	Teamwork	4.5 (4:4.5)	4.5 (4:4.5)	4.5 (4:5)	4.5 (4:4.5)
	Work satisfaction	3 (2.5:4)	3.5 (2.5:4)	4 (3.5:4)	3.5 (3:4)

	Stress (single item)	3 (3:4)	2 (2:3)	4 (3:4)	3.5 (3:4)
Motivation to change questionnaire		n=11	NA[a]	n=18	NA
	Social support in life	3 (3:3.5)	NA	3 (2.9:3)	NA
	Control in life	3 (3:3)	NA	3 (3:3)	NA
	Mastery in life	3 (3:3)	NA	3 (3:3)	NA
	Challenges in life	3 (3:3.5)	NA	3 (3:3)	NA
	Values	3 (2:3)	NA	3 (2:3)	NA
	Self-efficacy	4 (3:4)	NA	3 (3:4)	NA
	Self-confidence	4 (3:4)	NA	3 (3:4)	NA
	Coworker support	3 (3:3)	NA	3 (3:4)	NA
	Supervisory support	3 (2:3)	NA	3 (2:3)	NA
	Challenges in work	3.5 (3:4)	NA	3.5 (3:3.6)	NA
	Job control	3 (3:3)	NA	3 (2.8:3)	NA
	Goals	3.5 (3:4)	NA	3.5 (3:3.6)	NA

[a]N/A: not applicable.
