# Supplementary material for: Evaluation of a Web-Based Stress Management Program for Persons Experiencing Work-Related Stress in Sweden (My Stress Control): Randomized Controlled Trial
Source: JMIR Ment Health. 2021 Dec 9;8(12):e17314. doi: 10.2196/17314 (PMC8704112; doi:10.2196/17314)
Supplement: Multimedia Appendix 5 [file mental_v8i12e17314_app5.pdf]

Variables (min-max)	Drop outs intervention group	Drop outs wait list group
	Pre	Pre
	Median (IRQ, 25th percentile: 75th percentile)	Median (IRQ, 25th percentile: 75th percentile)
Perceived Stress Scale -14 (0-56)	n=36<br>27 (24.3:30.8)	n=25<br>28 (23.5:32.5)
Coping Self-Efficacy Scale (0-260)	n=33<br>141 (121:156.5)	n=23<br>142 (106:159)
Utrecht work engagement scale (0-6 for total/each sub scale)	n=33<br>3.9 (3.4:4.6)	n=20<br>3.8 (3.1:4.4)
Vigor	4 (3:4.5)	3.5 (2.7:4.3)
Dedication	4.33 (3.5:5)	4.2 (3.3:5)
Absorption	4 (3:4.5)	3.7 (3.1:4.3)
Brief COPE Questionnaire	n = 30	n = 18
Self-distraction (2-8)	5 (4.8:6)	(3:6)
Problem focused coping (4-16)	12 (11:12)	11 (10:12.3)
Avoidant coping (6-24)	9 (7.8:10)	(8:10)
Socially supported coping (6-24)	15 (13:18)	(12.8:19)
Emotional focused coping (8-32)	19.5 (16.8:21)	(15.3:20.5)
Self-blame (2-8)	5 (4:5)	5 (4:6)
Coping through emotional processing (1-4)	2.3 (2:2.8)	2.5 (2:3)
Coping through emotional expression (1-4)	1.9 (1.5:2.8)	2.4 (2:3)
QPS-Nordic 34+ (1-5 for each sub scale)	n = 32	n = 18
Quantitative demands	3.5 (3:4.4)	(3.5:4.1)
Demands on learning	2.5 (2:3)	2.5 (1.5:3)
Role clarity	3.5 (3.5:4)	(2.5:4.5)
Role conflicts (single item)	3 (2:3)	(3:4)
Positive challenges at work	4 (3.5:4.5)	(3.5:4.5)
Control over decisions	3 (2.5:3.5)	(2.5:3.1)
Control over working pace	3.5 (3:4)	3.3 (2:4.5)
Predictability over next month (single item)	4 (2:4)	(2:4)
Predictability (single item)	3 (2.3:4)	(2:5)
Experience of mastery (single item)	4 (3:4)	(3:4)
Support from employer	4 (3:4)	3.5 (3:4)
Support from colleagues (single item)	4 (3.3:4)	4 (3:5)
Support from friends and family (single item)	4 (3:4)	(2:4.3)
Social interaction (single item)	2.5 (2:3)	(2:4)
Encouraging leadership	3.5 (2.6:4)	3.3 (1.9:3.6)
Social climate	3.5 (3:4)	(3:4.5)
Innovative climate	4 (3:4)	3.3 (2.5:4)
Inequality	1 (1:2)	(1:2)

Personnel targets	3 (2.1:3.5)	2.3 (1.5:3.5)
Organizational culture and climate (single item)	3 (3:4)	3 (3:4)
Teamwork	4 (3.5:4.4)	(3:4.5)
Work satisfaction	4.5 (2.5:4)	(2:4)
Stress (single item)	3 (3:4)	3.5 (2.8:4)
Motivation to Change Questionnaire	n = 33	n = 23
Social support in life	3 (3:3)	(2:3)
Control in life	3 (3:3)	(3:3)
Mastery in life	3 (3:3)	(3:3)
Challenges in life	3 (3:3.5)	(3:3.5)
Values	3 (2.5:3)	(2:3)
Self-efficacy	3 (3:3)	(3:4)
Self-confidence	3 (3:4)	(3:4)
Co-worker support	3 (3:3)	(3:3)
Supervisory support	3 (2.5:3)	(2:3)
Challenges in work	3.5 (3:3.5)	(3:4)
Job control	3 (3:3)	(2:3)
Goals	3 (3:3.5)	3 (3:3.5)
